# Gut Microbiota Modulation in Asthma—An In Vitro Study

**DOI:** 10.3390/ph19010002

**Published:** 2025-12-19

**Authors:** Paulina Kleniewska, Paulina Natalia Kopa-Stojak, Rafał Pawliczak

**Affiliations:** Department of Immunopathology, Faculty of Medicine, Medical University of Lodz, Zeligowskiego 7/9, 90-752 Lodz, Poland

**Keywords:** intestinal microflora, asthma, *Bacteroides vulgatus*, *Clostridium perfringens*, *Parabacteroides distasonis*, *Ruminococcus albus*

## Abstract

**Objectives:** The aim of this work was to investigate whether *Bacteroides vulgatus* (BV), *Clostridium perfringens* (CP), *Parabacteroides distasonis* (PD), and *Ruminococcus albus* (RA) lysates modulate the secretion of IL-17, INF-γ, IL-2, and TGF-β 1 by human HT-29 cells, PBMCs, and monocytes (MON). **Results:** CP lysate significantly lowered IL-17 secretion by HT-29 cells vs. control (*p* < 0.05), but only at a dose of 100 µg. RA lysate reduced IL-17 secretion by HT-29 cells vs. control (*p* < 0.05), but only at a dose of 400 µg, whereas PD lysate significantly decreased IL-17 secretion by HT-29 cells vs. control (*p* < 0.05) at both doses. The secretion of IL-17 by PBMCs was significantly reduced after administration of BV and PD lysates (100 µg). BV and PD lysates (400 µg) also significantly decreased IL-17 secretion by MON vs. control (*p* < 0.05). The secretion of INF-γ by HT-29 cells was significantly lowered vs. control (*p* < 0.05) after administration of PD and CP lysates (400 µg). CP lysates (100 µg and 400 µg) also significantly reduced INF-γ secretion by MON compared with control (*p* < 0.05). The secretion of INF-γ by PBMCs was significantly reduced vs. control (*p* < 0.05) after administration of BV and CP lysates (400 µg). **Conclusions:** In PBMCs, HT-29 cells, and MON, INF-γ and IL-17 concentrations were significantly lowered by selected bacterial lysates in a dose-dependent manner. However, the low values detected in this experiment may not have an impact on systemic immune status.

## 1. Introduction

Asthma is a heterogeneous disease syndrome with a variable course and response to treatment [1]. Its prevalence has increased significantly in recent decades. In 2019, asthma affected almost 270 million people [2]. This number varies, but it is estimated to reach up to 300 million patients worldwide. Globally, the disease causes 1000 deaths per day [1]. Chronic inflammation of the bronchi is closely related to airway hyperreactivity and leads to recurrent clinical symptoms such as shortness of breath, wheezing, or a feeling of difficulty breathing. These symptoms occur with particular frequency at night and/or in the morning. The phenomenon of airway remodeling is associated with a progressive loss of functionality.

In recent years, numerous environmental factors have been identified as contributing to the development of this complex, etiological condition. One of them is the composition of the intestinal microflora. Microorganisms that inhabit the gut include mainly bacteria but also fungi and viruses. Many authors have demonstrated a link between microflora and the maintenance of the body’s homeostasis [3,4,5]. The human microbiota includes, among others, *Bacteroidetes* and *Firmicutes*, which constitute approximately 90% of its composition, as well as *Actinobacteria*, *Proteobacteria*, and *Verrucomicrobia*. The importance of a healthy microbiota was previously understood primarily in the context of digestion, the production of vitamin K and B vitamins, and protection against infections [6]. Moreover, it reduces the risk of developing digestive system diseases and even cardiovascular diseases [7].

An imbalance in the intestinal microflora caused by unfavorable changes in the diversity or abundance of microorganisms is called dysbiosis [8]. The most important risk factors for intestinal microbiota dysbiosis include breastfeeding or formula feeding, mode of delivery, diet, antibiotic use, and lifestyle. In infants born vaginally, a predominance of *Bifidobacterium*, *Escherichia*, *Bacteroides*, and *Parabacteroides* bacteria has been demonstrated. In infants delivered by cesarean section, the predominant bacteria are *Enterococcus*, *Staphylococcus*, *Streptococcus*, *Klebsiella*, *Enterobacter*, and *Clostridium* [5]. Even short-term administration of antibiotics (especially broad-spectrum antibiotics) has been shown to cause significant changes in children’s developing microflora, which may not fully recover within 12 months of completing treatment. *Bifidobacterium* spp. are primarily susceptible to antibiotics, resulting in their elimination and the promotion of pathogenic bacteria such as *Klebsiella* and *Enterococcus* spp. [9].

The aim of this study was to investigate the influence of gut bacteria—*B. vulgatus, C. perfringens*, *P. distasonis* and *R. albus* lysates—on the secretion of IL-17, INF-γ, IL-2 and TGF-β 1 by PBMCs, monocytes and HT-29 cells. The gut microbiome influences immune system maturation and inflammation (the “gut–lung axis”, or so-called gut–lung interconnectedness). Therefore, dysbiosis can lead to an overactive immune response and the development of inflammation in the airways. Understanding the potential mechanisms of action of selected gut microbiota species in the development of asthma is essential for the development of new preventive and therapeutic strategies based on the modulation of undifferentiated and unbalanced gut microbiota [10,11]. This is the first study in which these four bacterial strains were used to assess the secretion of IL-2, INF-γ, IL-17, and TGF-β1 by PBMCs, monocytes, and HT-29 cells. Our research was preceded by a pilot study [12], in which significant differences in intestinal microbiota species between control and asthma patients were identified using next-generation sequencing (NGS).

## 2. Results

### Evaluation of IL-17, IFN-γ, IL-2, and TGF-β 1 Concentrations

**Figure 1:**
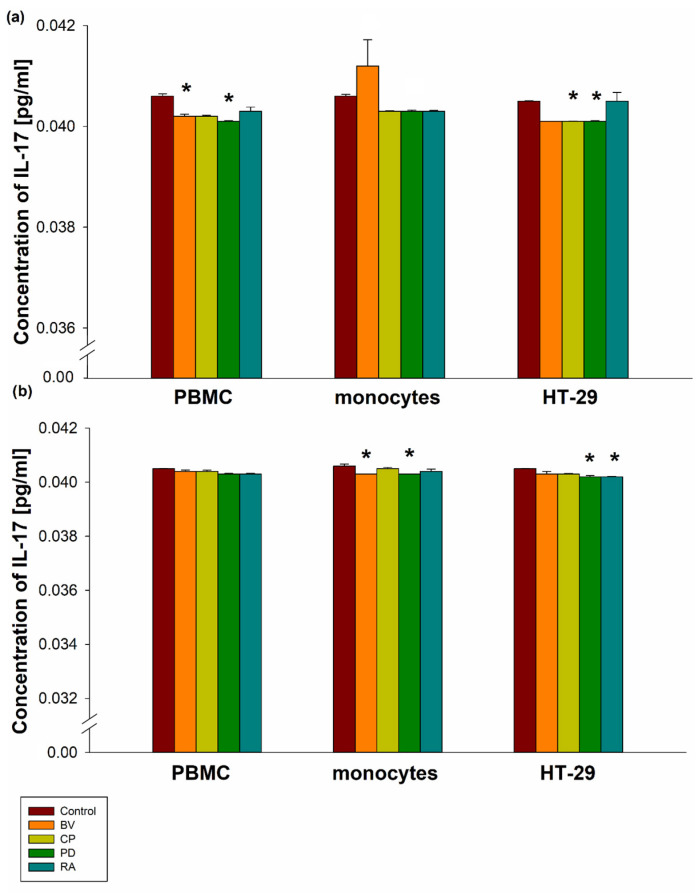
The effect of 100 µg (**a**) and 400 µg (**b**) bacterial lysates on IL-17 secretion by human peripheral blood mononuclear cells (PBMC), human monocytes, and human intestinal epithelial cells (HT-29). Human peripheral blood mononuclear cells were seeded in a 6-well plate at 2 × 10^6^ cells/well, and human monocytes and HT-29 cells at 0.5 × 10^6^ cells/well, and stimulated with 100 µg or 400 µg of bacterial lysates for 24 h. The concentration of IL-17 was measured by ELISA. The final results represent the mean of 3 independent repeats of human cell stimulation by bacterial lysates. Values are presented as mean ± SEM; * *p* < 0.05 vs. control group. Dunnett’s method was used. To increase the visibility of differences between tested lysates and the control, a broken x-axis bar graph was used. BV—*Bacteroides vulgatus*, CP—*Clostridium perfringens*, PD—*Parabacteroides distasonis*, RA—*Ruminococcus albus*.

*B. vulgatus* and *P. distasonis* lysates at a dose of 100 µg significantly decreased IL-17 secretion by PBMCs (0.04020401 ± 0.0000743 pg/mL for BV; 0.0401 ± 0.0000119 pg/mL for PD) compared to the control (0.0406 ± 0.0000477 pg/mL; *p* < 0.05). Lysates of *B. vulgatus*, *C. perfringens*, *P. distasonis*, and *R. albus* at a dose of 400 µg insignificantly decreased IL-17 secretion by PBMCs compared to the control. *B. vulgatus* lysate at a dose of 100 µg insignificantly increased IL-17 secretion, whereas at a dose of 400 µg it significantly decreased IL-17 secretion by MON (0.0412 ± 0.000517 pg/mL for 100 µg and 0.0403 ± 0.00000238 pg/mL for 400 µg) compared to the control (0.0406 ± 0.0000358 pg/mL for 100 µg and 0.0406 ± 0.0000691 pg/mL for 400 µg; *p* < 0.05). Moreover, *P. distasonis* lysates at a dose of 400 µg significantly decreased IL-17 secretion by MON (0.0403 ± 0.00000238 pg/mL) compared to the control (*p* < 0.05). Furthermore, *C. perfringens* lysate (100 µg) significantly decreased IL-17 secretion by HT-29 cells (0.0401 ± 0.00000715 pg/mL) compared to the control (0.0405 ± 0.00000715 pg/mL; *p* < 0.05). *P. distasonis* lysates at doses of 100 µg and 400 µg significantly decreased IL-17 secretion by HT-29 cells (0.0401 ± 0.0000119 pg/mL for 100 µg and 0.0402 ± 0.0000453 pg/mL for 400 µg) compared to the control (0.0405 ± 0.00000715 pg/mL for both doses; *p* < 0.05). A significant decrease in IL-17 secretion by HT-29 cells was also observed after stimulation with 400 µg of *R. albus* lysate (0.0402 ± 0.0000143 pg/mL) compared to the control (*p* < 0.05). However, no significant differences in IL-17 secretion were observed between the bacterial strains in PBMCs, MON, or HT-29 cells (Figure 1).

**Figure 2:**
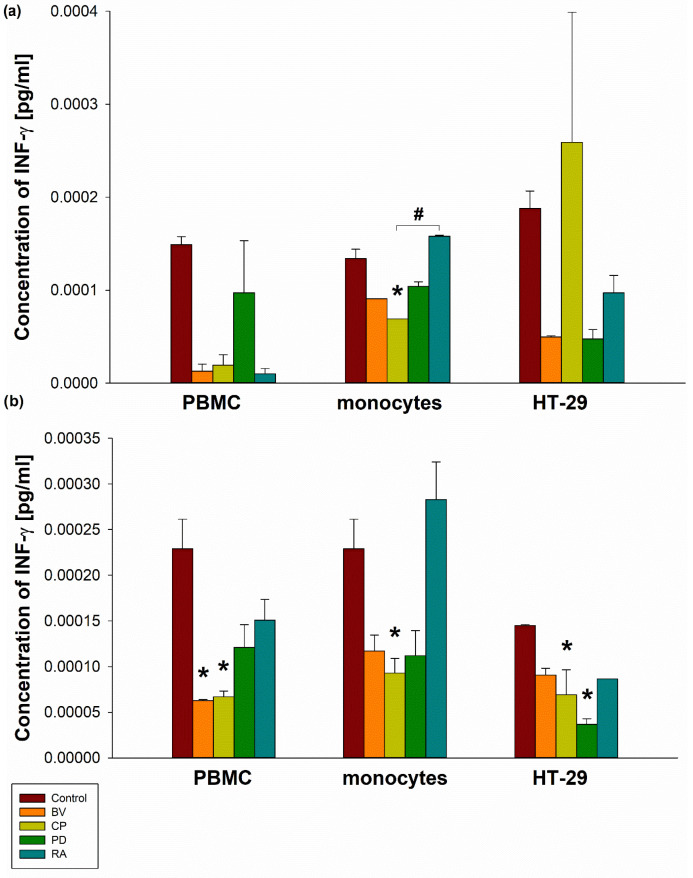
The effect of 100 µg (**a**) and 400 µg (**b**) bacterial lysates on INF-γ secretion by human peripheral blood mononuclear cells (PBMC), human monocytes, and human intestinal epithelial cells (HT-29). Human peripheral blood mononuclear cells were seeded in a 6-well plate at 2 × 10^6^ cells/well, and human monocytes and HT-29 cells at 0.5 × 10^6^ cells/well, and stimulated with 100 µg or 400 µg of bacterial lysates for 24 h. The concentration of INF-γ was measured by ELISA. The final results represent the mean of 3 independent repeats of human cell stimulation by bacterial lysates. Values are presented as mean ± SEM. Dunnett’s method was used; * *p* < 0.05 vs. control group. Additionally, based on the analysis of the Turkey test, significant differences were found in INF-γ secretion by monocytes stimulated with 100 µg of RA bacterial lysate (*p* < 0.05; rank difference: 36,000; q’ = 4648) compared to 100 µg of CP bacterial lysate (# *p* < 0.05 vs. CP). BV—*Bacteroides vulgatus*, CP—*Clostridium perfringens*, PD—*Parabacteroides distasonis*, RA—*Ruminococcus albus*.

*C. perfringens* lysate (100 µg) significantly decreased INF-γ secretion by human MON (0.0000691 ± 0.0000000001 pg/mL) compared to the control (0.000134 ± 0.00000998 pg/mL; *p* < 0.05). Moreover, a significant increase in INF-γ secretion was observed in human MON stimulated with 100 µg of *R. albus* lysate (0.000158 ± 0.00000125 pg/mL) compared to *C. perfringens* lysate at the same dose (*p* < 0.05). The control and 100 µg lysates of *B. vulgatus, C. perfringens, P. distasonis,* and *R. albus* showed similar INF-γ secretion by PBMCs and HT-29 cells. Furthermore, *C. perfringens* lysate at a dose of 400 µg significantly decreased INF-γ secretion by PBMCs (0.000067 ± 0.00000624 pg/mL), MON (0.0000929 ± 0.0000162 pg/mL), and HT-29 cells (0.0000691 ± 0.00274 pg/mL) compared to the control (0.000229 ± 0.0000324 pg/mL, 0.000229 ± 0.0000324 pg/mL and 0.000145 ± 0.00000125 pg/mL, respectively; *p* < 0.05). Moreover, *B. vulgatus* lysate at a dose of 400 µg significantly reduced INF-γ secretion by PBMCs (0.0000627 ± 0.00000125 pg/mL), as did *P. distasonis* lysate (400 µg) in HT-29 cells (0.0000367 ± 0.00000624 pg/mL) compared to the control (*p* < 0.05) (Figure 2).

**Figure 3:**
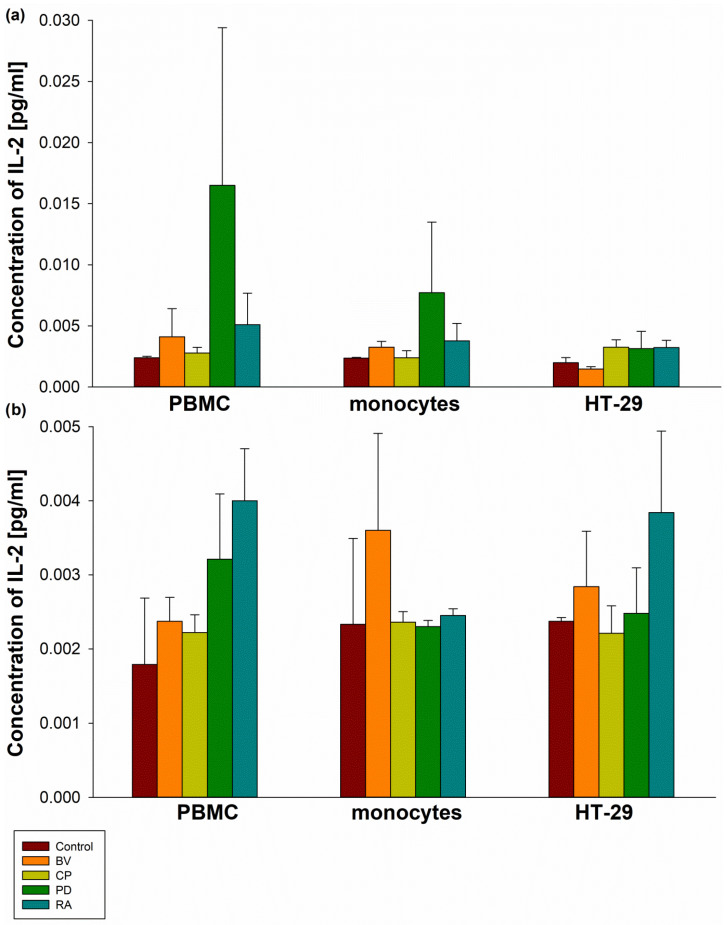
The effect of 100 µg (**a**) and 400 µg (**b**) bacterial lysates on IL-2 secretion by human peripheral blood mononuclear cells (PBMCs), human monocytes, and human intestinal epithelial cells (HT-29). Human peripheral blood mononuclear cells were seeded in a 6-well plate at 2 × 10^6^ cells/well, and human monocytes and HT-29 cells at 0.5 × 10^6^ cells/well, and stimulated with 100 µg (**a**) or 400 µg (**b**) of bacterial lysates for 24 h. The concentration of IL-2 was measured by ELISA. The final results represent the mean of 3 independent repeats of human cell stimulation by bacterial lysates. Values are presented as mean ± SEM; BV—*Bacteroides vulgatus*, CP—*Clostridium perfringens*, PD—*Parabacteroides distasonis*, RA—*Ruminococcus albus*.

**Figure 4:**
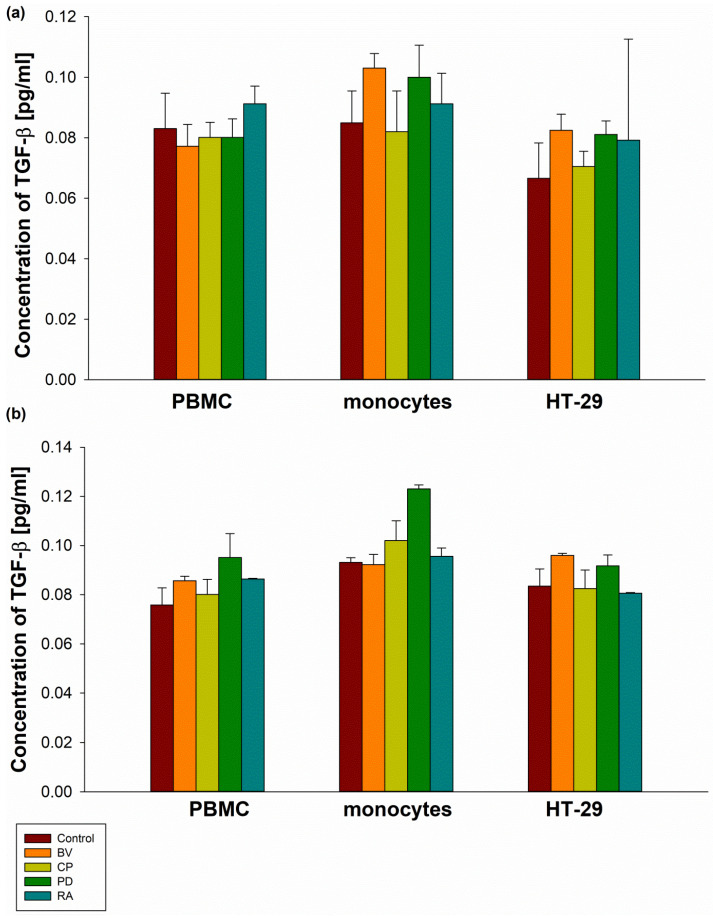
The effect of 100 µg (**a**) and 400 µg (**b**) bacterial lysates on TGF-β secretion by human peripheral blood mononuclear cells (PBMCs), human monocytes, and human intestinal epithelial cells (HT-29). Human peripheral blood mononuclear cells were seeded in a 6-well plate at 2 × 10^6^ cells/well, and human monocytes and HT-29 cells at 0.5 × 10^6^ cells/well, and stimulated with 100 µg or 400 µg of bacterial lysates for 24 h. The concentration of TGF-β was measured by ELISA. The final results represent the mean of 3 independent repeats of human cell stimulation by bacterial lysates. Values are presented as mean ± SEM; BV—Bacteroides vulgatus, CP—*Clostridium perfringens*, PD—*Parabacteroides distasonis*, RA—*Ruminococcus albus*.

No significant differences were observed between control samples and all bacterial lysates at concentrations of 100 µg and 400 µg in terms of IL-2 secretion by PBMCs, MON, and HT-29 cells (Figure 3). Similarly, no statistically significant differences were found in TGF-β secretion by PBMCs, MON, and HT-29 cells compared to the control. Furthermore, no significant differences in TGF-β secretion were observed between bacterial strains (Figure 4).

## 3. Discussion

Currently, topics related to gut microbiota and its association with the pathogenesis of diseases [13,14,15,16] are very popular among both researchers and patients. The microbiota has a profound impact on many diseases and also plays an important role in their prevention. Dysbiosis is an intestinal imbalance caused by unfavorable changes in the diversity or abundance of microorganisms [8]. Researchers suggest that the first three years of life are crucial for the development of a healthy microbiota. Microbiome diversity may change toward adult levels by the age of five, although this remains under investigation, suggesting that this period may be crucial for health and disease prevention [17,18]. Moreover, Thorburn et al. [19] demonstrated that asthma is a developmental disease influenced by the mother’s diet and bacterial metabolites. Scientists have reported that high-fiber or acetate feeding led to marked suppression of allergic airway disease (a model for human asthma) by enhancing T-regulatory cell numbers and function. On the other hand, a recent study by Lin et al. [20] did not find statistically significant differences in the concentration of short-chain fatty acids (SCFAs)—acetate, propionate, butyrate, valerate, isobutyrate, and isovalerate—at 20th or 28th week of gestation in the plasma of mothers whose children developed asthma versus those whose children did not. Arrieta et al. [21] reported that children who developed atopic asthma had significantly decreased numbers of bacteria from the genera *Faecalibacterium*, *Lachnospira*, *Veillonella*, and *Rothia* at 3 months of age. The same investigators [22] reported that increased relative abundance of *Bacteroides* and *Streptococcus* species and decreased abundance of *Ruminococcus gnavus* and *Bifidobacterium* species at 3 months of age are associated with a higher risk of developing asthma by 5 years of age.

Commensal bacteria enhance the immune response by producing immunomodulatory compounds such as SCFAs or microbe-associated molecular patterns. The authors of [23] demonstrated that butyrate reduces inflammation by decreasing the production of IFN-γ, IL-1, IL-2, IL-12, and TNF-α while simultaneously increasing the production of IL-10. It also promotes Treg cell differentiation [24]. SCFAs also lower the pH of the intestinal environment, inhibiting the growth of many pathogens. Butyrate can limit the growth of several *C. difficile* strains [25].

Modulation of disturbed bacterial microflora is particularly important for the prevention and treatment of microbiome-related diseases, including asthma [26]. Th2-high asthma may be indicated by elevated levels of FeNO, IgE, and eosinophils [27,28,29]. Importantly, in moderate to severe asthma, only one third of cases are caused by Th2-type inflammation [30]. Neutrophil-dominated or pauci-granulocytic inflammation with high levels of IFN-γ, IL-17A/F, and IL-17A/IL-22 cytokines released from Th1, Th17, or type 3 innate lymphoid cells characterizes the Th2-low endotype [31,32]. The inflammatory mediators of this more severe endotype include IL-1β, IL-6, TNF-α, IFN-γ, and IL-17 [28]. Potential biomarkers of asthma with low Th2 cell expression may include IL-6, neutrophils, or matrix metallopeptidase 9, although researchers have not confirmed that any represent all phenotypic subgroups of this endotype [33,34]. The authors describe that commensal gut flora induces differentiation of specific CD4+ T cell subtypes [35]; *Bacteroides fragilis* influences the development of systemic Th1 cells [36] and local IL-10-producing Tregs [37]; and indigenous *Clostridium* species induce Tregs in the colon [38].

Our previous results [39,40] indicated that *R. albus* and *P. distasonis* lysates at a dose of 400 µg significantly increased IL-5 secretion by PBMCs vs. control (*p* < 0.05). Furthermore, *B. vulgatus*, *C. perfringens*, *P. distasonis*, and *R. albus* at 100 µg significantly increased IL-8 secretion by PBMCs, as did *B. vulgatus*, *P. distasonis*, and *R. albus* lysates at 100 µg in monocytes vs. control (*p* < 0.05). Importantly, *C. perfringens*, *B. vulgatus*, *P. distasonis*, and *R. albus* lysates at 100 µg significantly decreased IL-13 secretion by PBMCs vs. control (*p* < 0.001). IL-6 secretion by PBMCs and monocytes increased significantly (*p* < 0.05) after administration of *C. perfringens* and *P. distasonis*. *B. vulgatus*, *C. perfringens*, and *P. distasonis* lysates (100 μg) also significantly increased IL-10 secretion by PBMCs vs. control (*p* < 0.05).

Our present findings indicate that in PBMC, HT-29, and MON cells, INF-γ and IL-17 concentrations were significantly reduced by selected bacterial lysates in a dose-dependent manner, although cytokine concentrations were extremely low. Our results suggest that *C. perfringens* significantly reduced IL-17 secretion by HT-29 compared to the control at 100 µg, and *R. albus* reduced its secretion at 400 µg. *P. distasonis* lysates significantly reduced IL-17 secretion by HT-29 at both doses. IL-17 secretion by PBMCs was significantly reduced after administration of *B. vulgatus* and *P. distasonis* lysates (100 µg) compared to the control group. *B. vulgatus* and *P. distasonis* also significantly reduced the secretion of this cytokine by monocytes, but only at 400 µg. However, *B. vulgatus* lysates at a dose of 100 µg slightly increased the generation of the discussed interleukin by monocytes, though not significantly.

Th17 cells and their cytokine IL-17 are associated with the development of severe asthma [41]. Severe asthma is generally characterized by increased levels of IL-17 mRNA and protein in lung tissue, induced sputum, and serum [42,43,44]. The goal of treatment should be to modulate the action or target the pathways that contribute to inflammation. Interestingly, Kim et al. [45] demonstrated that simultaneous targeting of IL-17 and IL-13 pathways may result in more effective treatment because neutralization of both IL-17A and IL-13 inhibits neutrophilia, eosinophilia, mucus hyperplasia, and AHR in a mouse model of asthma.

IL-17 acts synergistically with other activators of STAT1 (IFN-γ), STAT6 (IL13), NF-κB (TNF-α), and SMAD (TGF-β) [46]. The authors of [47] reported that glucocorticosteroids have no effect on IL-17 expression in asthmatic animals. On the other hand, Wu et al. [48] described that after glucocorticosteroid treatment, the sputum percentage of eosinophils, neutrophils, and IL-17 levels decreased significantly in all asthmatics (all *p* < 0.01). Additionally, steroid treatment itself intensifies dysbiosis [49].

Studies have shown that respiratory microorganisms can directly or indirectly induce Th17 cell differentiation and the production of related cytokines, triggering airway hyperresponsiveness (AHR) [50]. Several years ago, the authors of [51] described that colonization by *Staphylococcus aureus* induces a Th17 response with elevated IL-17 levels, while colonization by *Moraxella catarrhalis* and *Haemophilus influenzae* induces a mixed Th1/Th2/Th17 response. However, other researchers [52] have reported that shifts in the lung microbiome from the dominant phyla *Firmicutes* and *Gammaproteobacteria* toward *Bacteroidetes* may be a key factor in inducing Treg cells via PD-L1. Recently, Liu et al. [53] reported that *S. boulardii* reduces symptoms of allergic asthma by restoring gut microbiome and metabolic homeostasis by upregulating METTL3 expression in an m6A-dependent manner. Song et al. [54] described that *C. perfringens* significantly increases Th17/Treg (percentage of Th17 cells in ileum and blood, Th17/Treg ratio, IL-17 and IL-17/IL-10 ratio in blood). The study [55] confirmed the disruption of the lung microbiome in neutrophilic asthma. Importantly, a longer course of asthma, a worse predicted FEV1%, and higher proportions and numbers of neutrophils in sputum are associated with the development of steroid-resistant asthma. Induced sputum is dominated by *Moraxella catarrhalis*, *Haemophilus*, or *Streptococcus* [56]. Other authors [57,58] also described the involvement of IL-17 in refractory neutrophilic asthma associated with disturbances in the lung microbiome. The study [59] confirmed that several families in *Proteobacteria* correlate positively with the expression of Th17-related genes. The role of IL-17 in neutrophilic asthma with a disrupted microbiome was also confirmed by Yang et al. [60]. *S. pneumoniae* in infancy increased the risk of allergic airway disease in adulthood, with elevated Th17/IL-17 levels and neutrophil accumulation. Neutralization of IL-17 significantly reduced neutrophil recruitment, attenuated airway inflammation, and reduced AHR.

Our results show that INF-γ secretion by HT-29 was significantly lower than in the control group after administration of *P. distasonis* lysates (400 µg). *C. perfringens* lysates (400 µg) also significantly reduced INF-γ secretion by monocytes compared with the control group. INF-γ secretion by PBMCs was significantly lower than in the control group after *B. vulgatus* administration, but only at the 400 µg lysate dose.

Th1 cells, through the production of interferon gamma, induce cell-mediated immunity and inhibit IL-4 secretion by Th2 cells, as well as Th2 cell proliferation in vitro [61]. In mice, this cytokine blocks the effects of Th2-induced airway eosinophilia and AHR [62]. Th1 cells also promote immunoglobulin class switching to IgG2a [63]. INF-γ, together with IL-1 and TNF-α, increases CD38 expression in airway smooth muscle cells, which may lead to increased intracellular Ca^2+^ signaling and induce AHR [64].

In the course of asthma, large amounts of IgE antibodies are produced by B lymphocytes, and the IFN-γ/IL-4 (Th1/Th2) ratio decreases [65]. The results of Al-Daghri et al. [66] also confirmed the occurrence of a significant decrease in INF-γ concentration in asthmatic children. Furthermore, this cytokine promotes neutrophil recruitment in the presence of IL-17 [67,68]. The authors described that CP increased IFN-γ relative expression levels in necrotic enteritis birds [69]. Other researchers have reported similar results [70]. The concentration of this cytokine decreased after the use of functional oligosaccharides [71].

This paper shows that *B. vulgatus, C. perfringens, P. distasonis* lysates, and *R. albus* (400 µg) only slightly increased IL-2 secretion by PBMCs compared to control. No statistically significant differences were noted in the secretion of this interleukin. Dysregulation of IL-2 family cytokines has been implicated in the pathogenesis of asthma [72]. Authors [73] noted inhibition of IL-2 secretion by PBMCs treated with different doses of LPS from *Bacteroides fragilis*. The study by Hata et al. [74] showed increased colonization of *Bacteroides* spp. and increased IL-2 secretion in HLA-B27/beta2-microglobulin transgenic rats. Krakauer et al. [75] did not describe the significant effect of different doses of CP enterotoxin on IL-2 secretion by PBMCs. These results were confirmed in in vitro and in vivo models presented by Wallace et al. [76]. CPE exposure had no effect on IL-2 secretion by macrophages from J774A and Swiss Webster mice. Daneshmanda et al. [77] reported that CP induces an enhanced T cell response (including activation of CD4+ and CD8+ T cells) and increases IL-2 secretion by these cells in the chicken jejunum. Similar results were obtained by [78], who described increased IL-2 expression in the duodenum and jejunum after three days of oral CP administration. However, a study by Fasina and Lillehoj [79] showed reduced IL-2 gene expression in the jejunum and cecum. Chu et al. [80] found that reduced IL-2 levels were associated with reduced *Ruminococcus* abundance in patients with systemic lupus erythematosus. Other authors [81] have shown that probiotic supplementation in pregnant Bama mini piglets increases the number of *Ruminococcus* and *Bacteroides* bacteria in the feces and is also associated with a decrease in the concentration of IL-2 in plasma. A study on an animal model (sheep) confirmed the association between increased levels of IL-2 and TGF-β and low *Ruminococcus* counts in the jejunal mucosa [82].

Our results suggest that *C. perfringens, B. vulgatus,* and *P. distasonis* lysates only slightly decreased TGF-β secretion by PBMCs compared to control. No statistically significant differences were noted in the secretion of this cytokine. TGF-beta 1 also plays a role in the pathogenesis of bronchial asthma [83,84,85]. Some authors suggest a correlation between higher TGF-β concentrations and a more severe course of asthma [86].

Koh et al. [87] reported that male A/J mice exposed to *P. distasonis* showed a significant increase in TGF-β gene expression (185%; *p* < 0.001) as well as TGF-β secretion (47–145%; *p* < 0.05) compared to those not exposed. Interestingly, a study [88] conducted on healthy female Holstein cattle showed lower levels of *Bacteroidaceae*, *Eubacterium,* and *Bifidobacterium* in older animals, but higher levels of inflammatory cytokines, including TGF-β (*p* < 0.001), compared to younger animals. Sanfilippo et al. [89] reported increased secretion of TGF-β by different human intestinal epithelial cell lines exposed to *Bacteroides fragilis* toxin. Dieleman et al. [90] reported a significant difference in TGF-β levels between B27 TG rats monoassociated with BV for four weeks and those treated with vancomycin and imipenem, as well as Lactobacillus rhamnosus GG and Lactobacillus plantarum 299v. Shojadoost et al. [78] observed increased TGF-β gene expression in the jejunum and duodenum of broilers orally infected with CP compared to the control group. However, Fasina and Lillehoj [79] showed that CP infection caused a decrease in TGF-β expression. Liu et al. [91] reported a reduction in TGF-β gene expression in a mouse model of acute liver failure and a simultaneous increase in *Bacteroidetes* bacteria and a decrease in *Ruminococcus* bacteria [92].

Unfortunately, there are not enough research projects with a similar methodology evaluating the use of the discussed bacterial strains in the context of asthma. The results remain inconclusive because the research techniques have not yet been systematized. Further research in this area is necessary to provide additional information on detailed therapy regimens, dosage, and the selection of specific bacterial strains for specific patient groups. In the future, researchers should focus on assessing not only the efficacy but also the safety of the species/strains used.

## 4. Materials and Methods

### 4.1. Selected Strains of Intestinal Microflora and Selected Human Cell Lines

As described previously [40,41], the study included 4 types of bacteria: *Clostridium perfringens* (ATCC 13124), cultivated in ATCC 2107 Modified Reinforced Clostridial Medium (LGC Standards, Teddington, UK); *Parabacteroides distasonis* (ATCC 8503), harvested in ATCC 1490 Modified Chopped Meat Medium (LGC Standards, Teddington, UK); *Bacteroides vulgatus* (ATCC 8482), cultivated in ATCC 2107 Modified Reinforced Clostridial Medium (LGC Standards, Teddington, UK); and *Ruminococcus albus* (ATCC 27210), harvested in ATCC 158 RGCA Medium (LGC Standards, Teddington, UK). Thawed vials of bacterial strains were aseptically transferred to 5–6 mL tubes containing pre-reduced media and cultivated for 24–48 h in 37 °C under anaerobic conditions. The bacterial cultures were transferred to new broth tubes with fresh medium every 24–48 h. In addition, ATCC 260 Trypticase soy agar with 5% sheep blood was used as a selection medium to check for contamination.

In this study, commercially purchased human cell lines were used. Human colon adenocarcinoma (HT-29) cells (cultivated in McCoy’s 5A (ATCC #30-2007) supplemented with fetal bovine serum (ATCC #30-2020)) were obtained from LGC Standards (Teddington, UK), and human peripheral blood mononuclear cells (PBMCs) (harvested in Mononuclear Cell Medium (Sigma-Aldrich #C-28030)) were obtained from Sigma-Aldrich (Saint Louis, MO, USA). Human monocytes (MON) (harvested in RPMI-1640 medium (Sigma-Aldrich #R8758) supplemented with 10% FBS (Sigma-Aldrich #F4135)) were isolated from PBMCs. To isolate human MON, PBMCs were incubated in T75 flasks for 2 h under standard conditions (37 °C, 5% CO_2_, 90% humidity), allowing temporary adhesion to separate MON from other PBMCs. Remaining cells were removed by washing three times with warm RPMI-1640 medium (Sigma-Aldrich, Saint Louis, MO, USA). Cell culture media were supplemented with the combination of penicillin/streptomycin (Sigma-Aldrich, #P4333). Cells viability was assessed using trypan blue and calculated using the formula: number of live cells/(number of live + dead cells) × 100 (presented as a percentage).

### 4.2. Preparation of Bacterial Strain Lysates and Stimulation of Human Cell Lines with Lysates

Bacterial cultures were transferred to 2 mL Eppendorf tubes and centrifuged (5 min, 10,000 rpm, 4 °C). Bacterial cell pellets were suspended in 1 mL of distilled water and disrupted using an ultrasonic disintegrator. Samples were then centrifuged (5 min, 10,000 rpm, 4 °C), the supernatants were transferred to new tubes, and stored at −20 °C.

MON and human HT-29 cells were seeded at a density of 0.5 × 10^6^ cells/well, and PBMCs were seeded at a density of 2 × 10^6^ cells/well in a 6-well plates. Cells were stimulated with lysates of *C. perfringens*, *R. albus*, *B. vulgatus*, and *P. distasonis* at concentrations of 100 µg and 400 µg for 24 h. Controls consisted of cells incubated in fresh culture medium or with 25 µg/mL dexamethasone. After 24 h of stimulation of HT-29 cells, PBMCs, and monocytes with bacterial lysates, the samples were centrifuged (5 min, 1500 rpm, 4 °C), and the supernatants were transferred to new collection tubes and stored at −20 °C.

### 4.3. Analysis of Selected Cytokine Secretion by ELISA Assay

To analyze the secretion of IL-17 (RAB0262), IFN-γ (RAB0222), IL-2 (RAB0286), and TGF-β 1 (RAB0460) by HT-29 cells, PBMCs, and monocytes following incubation with *C. perfringens*, *R. albus*, *B. vulgatus,* and *P. distasonis* lysates, enzyme-linked immunosorbent assays were performed. All commercially available ELISA tests were purchased from Merck KGaA (Darmstadt, Germany).

To assess the concentrations of individual cytokines, 100 µL of standards and 100 µL of each sample were added to duplicate wells of 96-well plates and incubated for 2.5 h at room temperature with gentle shaking. Plates were washed four times with 300 µL of wash solution per well. Then, 100 µL of detection antibody was added to each well and incubated for 1 h at room temperature with gentle shaking. After four additional washes (300 µL/well), 100 µL of streptavidin solution was added to each well and incubated for 45 min at room temperature with gentle shaking. Plates were washed again, followed by the addition of 100 µL of substrate reagent and incubation for 30 min at room temperature in the dark with gentle shaking. Finally, 50 µL of stop solution was added, and absorbance was measured at 450 nm.

### 4.4. Statistical Analysis

Results were presented as mean ± SEM. The statistical analysis was performed using the Kruskal–Wallis H test (one-way ANOVA on ranks), followed by the Tukey test and Dunnett’s method. A *p* value < 0.05 was considered statistically significant.

## 5. Conclusions

This study did not clearly confirm that intestinal bacteria lysates (*B. vulgatus*, *C. perfringens*, *P. distasonis,* or *R. albus*) can modulate the INF-γ and IL-17 in PBMC, HT-29, and MON cells. It appears that the extremely low values detected in this experiment will not have a significant impact on systemic immunity.

## Data Availability

The original contributions presented in this study are included in the article. Further inquiries can be directed to the corresponding author.

## Abbreviations

AHR	airway hyperresponsiveness
BV	*Bacteroides vulgatus*
CP	*Clostridium perfringens*
MON	monocytes
PD	*Parabacteroides distasonis*
PBMCs	peripheral blood mononuclear cells
RA	*Ruminococcus albus*
SCFAs	short-chain fatty acids
